# Effect of optically modified polyethylene terephthalate fiber socks on chronic foot pain

**DOI:** 10.1186/1472-6882-9-10

**Published:** 2009-04-22

**Authors:** Robyn MB York, Ian L Gordon

**Affiliations:** 1 Division of Vascular Surgery, Department of Surgery, University of California Irvine Medical Center, Orange CA, USA

## Abstract

**Background:**

Increasing experimental and clinical evidence suggests that illumination of the skin with relatively low intensity light may lead to therapeutic results such as reduced pain or improved wound healing. The goal of this study was to evaluate prospectively whether socks made from polyethylene terephthalate (PET) incorporating optically active particles (Celliant™) ameliorates chronic foot pain resulting from diabetic neuropathy or other disorders. Such optically modified fiber is thought to modify the illumination of the skin in the visible and infrared portions of the spectrum, and consequently reduce pain.

**Methods:**

A double-blind, randomized trial with 55 subjects (38 men, 17 women) enrolled (average age 59.7 ± 11.9 years), 26 with diabetic neuropathy and 29 with other pain etiologies. Subjects twice completed the Visual Analogue Scale (VAS), Brief Pain Inventory (BPI), McGill Pain Questionnaire (MPQ), and SF-36 a week apart (W_1+2_) before receiving either control or Celliant™ socks. The same questionnaires were answered again one and two weeks (W_3+4_) later. The questionnaires provided nine scores for analyzing pain reduction: one VAS score, two BPI scores, five MPQ scores, and the bodily pain score on the SF-36. Mean W_1+2 _and W_3+4 _scores were compared to measure pain reduction.

**Results:**

More pain reduction was reported by Celliant™ subjects for 8 of the 9 pain questions employed, with a significant (p = 0.043) difference between controls and Celliant™ for McGill question III. In neuropathic subjects, Celliant™ caused more pain reduction in 6 of the 9 questions, but not significantly. In non-neuropathic subjects 8 of 9 questions showed more pain reduction with the Celliant™ socks.

**Conclusion:**

Socks with optically modified PET (Celliant™) appear to have a beneficial impact on chronic foot pain. The mechanism could be related to the effects seen with illumination of tissues with visible and infrared light.

**Trial Registration:**

ClinicalTrials.gov NCT00458497

## Background

Celliant™ is a polymer fabric constructed from polyethylene terephthalate (PET) yarn containing optically active particles – a proprietary mixture of natural and inorganic materials – which scatter and reflect visible and near infrared light. Garments constructed with such optically modified fibers are thought to influence transmission and reflectance of electromagnetic energy into underlying tissue and skin. Numerous anecdotal reports from patients with a variety of chronic pain syndromes indicate that wearing Celliant™ garments for even a few days leads to dramatic improvement or complete resolution in subjective pain. We report here the results of a prospective, blinded study designed to substantiate the ability of Celliant™ socks to ameliorate chronic pain resulting from diabetic neuropathy and other disorders of the foot.

## Methods

This study was conducted at the Veterans Administration Medical Center Long Beach and approved by the local ethics board. All subjects reviewed an Informed Consent document and gave consent prior to enrolment. Fifty-five subjects (38 men, 17 women, age 59.7 ± 11.9) were enrolled, 26 with diabetic neuropathy and 29 with other causes of foot pain. Inclusion criteria included age ≥ 21, foot pain for at least six months, and a score of ≥ 3 on question III of the McGill Short Form Pain Questionnaire (MPQ) at screening. Subjects with diabetic neuropathy (DPN) had a minimum of 2/6 anesthetic points by Semmes-Weinstein filament testing on one foot. Subjects without DPN had 0/6 anesthetic points. Exclusion criteria included severe peripheral arterial disease (PAD) (ABI < 0.5), inability to ambulate, chronic ulceration, and severe psychiatric disorders. For subjects without DPN, etiologies included arthritis, erythromelalgia, Parkinson's disease, and PAD (Table 1). The most common foot pain etiology was arthritis.

**Table 1:** Pain etiologies in non-DPN subgroup

**Etiology**	**Celliant™**	**Control**
Arthritis	45%	40%

Edema	7%	0%

Erythromelalgia	0%	7%

Parkinson's Disease	0%	12%

PAD	0%	7%

Plantar Fasciitis	0%	7%

Previous Chemotherapy	7%	0%

Previous Surgery	7%	7%

Other Causes	36%	20%

At screening (week 1) subjects underwent physical examination including monofilament testing and completed a series of four questionnaires (Visual Analogue Scale [1] [VAS], Brief Pain Inventory [2,3] [BPI], MPQ [4], and SF-36 Quality of Life Inventory [5]). Only the bodily pain score from the SF-36 questionnaire was used to assess pain responses. Subjects completed the same questionnaires a week later (week 2) and were given 3 pairs of socks in a closed container and asked to wear them exclusively for the next two weeks. One (week 3) and two weeks (week 4) later they filled out the same panel of questions. Controls received socks made from standard 1.2 denier PET fabric, while the Celliant™ group received otherwise identical socks except PET containing Celliant™ particles was used to fashion the bottom (plantar) half of the garments. Both study personnel and subjects were blinded to the treatment assigned.

As the MPQ has 5 components (Ia, Ib, Ia+b, II, III) and the BPI 2 components (Pain Severity, Pain Interference), a total of 9 questions assessing pain were analyzed to measure subjects' responses. Mean scores for individual questions were calculated for the first two (W_1+2_) and final two visits (W_3+4_). Differences between W_1+2 _and W_3+4 _scores reflected changes in perceived pain resulting from wearing socks. Non-parametric two tailed t-test analysis (Mann-Whitney) was used to compare changes in scores [(mean W_1+2_) - (mean W_3+4_)] for individual questions reported by control and Celliant™ subjects. Analyses were performed on all 55 subjects as well as DPN and non-DPN subgroups.

## Results

Control and Celliant™ subjects had comparable age and gender distributions upon entry into the study (Table 2). Except for the BPI questions in the non-DPN subjects, there were no significant (p < 0.05) differences in the mean scores for individual questions at screening.

**Table 2:** Subject Characteristics Prior to Treatment

	**Demographics**		**McGill**				
**All Subjects**	**Age**	**% male**	**I-a**	**I-b**	**I-a+b**	**II**	**III**

Celliant™	57.7 ± 11.8	70%	1.2 ± 0.8	0.6 ± 0.7	1.9 ± 1.5	4.7 ± 2.4	2.6 ± 1.0

Control	61.6 ± 11.8	68%	1.3 ± 0.7	1.1 ± 1.0	2.4 ± 1.6	5.4 ± 2.8	3.1 ± 1.1

**DPN group**							

Celliant™	63.0 ± 7.7	85%	1.2 ± 0.9	0.6 ± 0.7	1.9 ± 1.5	5.1 ± 2.6	2.7 ± 1.1

Control	63.9 ± 11.0	77%	1.4 ± 0.7	1.2 ± 1.1	2.5 ± 1.7	5.2 ± 2.9	2.9 ± 0.9

**Non-DPN group**							

Celliant™	52.7 ± 13.1	57%	1.2 ± 0.8	0.6 ± 0.8	1.9 ± 1.5	4.4 ± 2.3	2.4 ± 0.9

Control	59.5 ± 12.3	60%	1.3 ± 0.8	1.1 ± 1.0	2.3 ± 1.6	5.6 ± 2.8	3.3 ± 1.2

	**Brief Pain Inventory**						

**All Subjects**	**Pain Severity**		**Pain Interference**	**VAS**		**SF-36: Bodily Pain**	

Celliant™	4.2 ± 2.4		5.8 ± 2.4	37.8 ± 8.1		4.2 ± 2.4	

Control	5.5 ± 2.6		6.4 ± 1.8	34.6 ± 7.8		5.5 ± 2.6	

**DPN group**							

Celliant™	4.9 ± 2.0		4.7 ± 2.5	5.9 ± 2.4		34.2 ± 7.4	

Control	5.1 ± 2.3		5.5 ± 2.9	6.1 ± 1.9		36.1 ± 7.5	

**Non-DPN group**							

Celliant™	3.9 ± 1.9*		3.8 ± 2.3*	5.8 ± 2.5		40.8 ± 7.7	

Control	5.3 ± 1.6*		5.6 ± 2.3*	6.6 ± 1.8		33.3 ± 8.1	

*denotes significant (p < 0.05) differences between Celliant™ and Control subjects.

Both control and Celliant™ subjects reported decreased subjective pain after wearing socks for every question based on comparing W_1+2 _scores to W_3+4 _scores (see Figures). The differences between W_1+2 _and W_3+4 _scores were significant (p < 0.05, Mann Whitney) in 6 of 9 questions for Celliant™ subjects and in 4 of 9 questions for controls. Improvement in pain scores before and after treatment is characteristic of a strong placebo effect generally seen in pain studies. For most questions, however, more improvement was reported by the entire Celliant™ group compared to the entire control group based on the magnitude of differences in [W_1+2 _- W_3+4_] scores.

**Figure 1:**
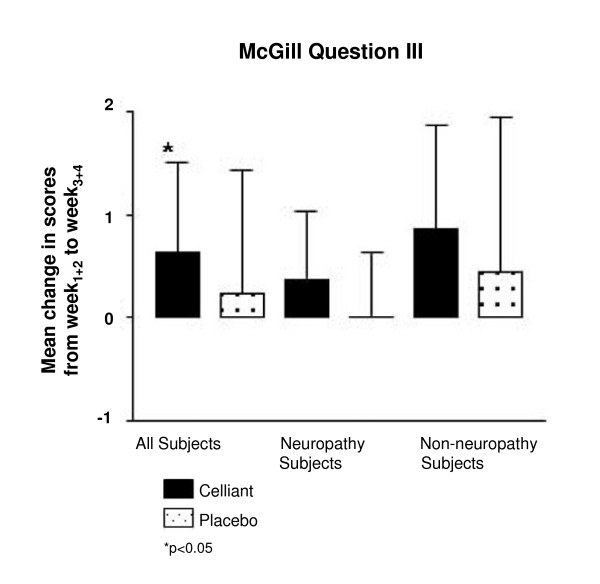
**Results of McGill Question III**. The difference between mean W_1+2 _and mean W_3+4 _scores is depicted. Solid bars report Celliant™ and stipled bars report control subjects. *p < 0.05.

Questions Ia and Ib of the MPQ rate the intensity of various aspects of pain: Question Ia rates 11 sensory aspects of pain such as throbbing or cramping as absent, mild, moderate, or severe. Question Ib similarly rates four affective dimensions (e.g., fearful). Question II is a simple scale where the intensity of present pain is marked on a line. Question III rates overall pain on a 0 (absent) to 5 (excruciating) scale. For control and Celliant™ groups, little difference between the improvements in mean scores for questions Ia, Ib, and Ia+b were found. The Celliant™ group demonstrated an improvement in pain for questions Ia (0.34 vs. 0.20, p = 0.634) and Ia+b (0.52 vs. 0.50, p = 0.829). For question Ib controls, however, showed a modestly greater reduction in pain compared to the Celliant™ subjects (0.17 vs. 0.10, p = 0.405). In question III (Figure 1), pain reduction for Celliant™ subjects was significantly greater (0.50 versus 0.00) than for controls (p = 0.043). For subjects with DPN, Celliant™ subjects reported more pain reduction in question Ia (0.22 vs. 0.19, p = 0.978), whereas controls reported more reduction in pain for questions Ib (0.06 vs. -0.01, p = 0.566), and Ia+b (0.45 vs. 0.21, p = 0.587). In question II, 19% more improvement was seen with Celliant™ in DPN subjects (p = 0.703). For question III, DPN subjects wearing Celliant™ socks showed a reduction of pain of 0.50 versus 0.00 in controls (p = 0.148). The Celliant™ group displayed minor improvements in pain scores for questions Ia (0.44 vs. 0.22, p = 0.571) and Ia+b (0.79 vs. 0.55, p = 0.896) in non-DPN subjects. Controls demonstrated more improvement for question Ib (0.28 vs. 0.20, p = 0.615) in this group. For question II in the non-DPN subjects, a nearly two-fold difference in pain reduction was seen with Celliant™ socks compared to controls (1.20 vs. 0.65, p = 0.371). For question III in non-DPN subjects, more reduction in pain was reported with Celliant™ (0.50 versus 0.00, p = 0.154).

**Figure 2:**
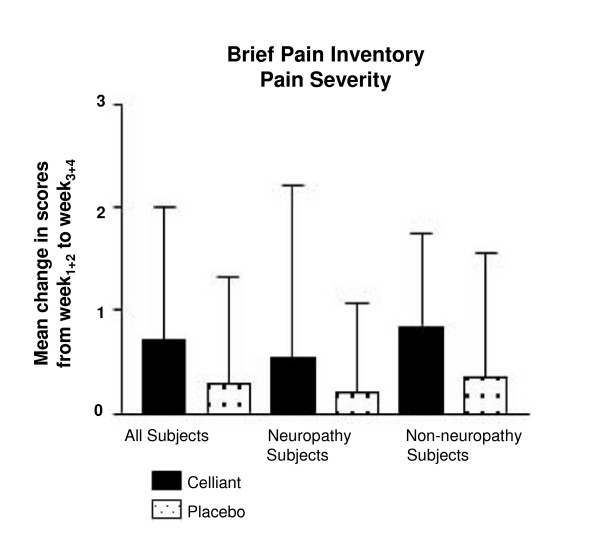
**Results of the Brief Pain Inventory – Pain Severity**. The difference between mean W_1+2 _and mean W_3+4 _scores is depicted. Solid bars report Celliant™ and stipled bars report control subjects.

Two scores are derived from the BPI. The severity score rates pain over the previous 7 days, past 24 hours, and present between 0 (absent) and 10 (worst possible). The interference score measures interference with activities such as walking and working from 0 (none) to 10 (complete). Celliant™ subjects reported 30% more reduction in severity compared to controls (p = 0.077, Figure 2). For interference, the Celliant™ group reported 18% more reduction than controls (p = 1.000). Celliant™ subjects with DPN reported a reduction in pain severity of 0.75 compared to 0.50 in the controls (p = 0.211) (Figure 2). For interference, controls demonstrated a greater reduction compared with the Celliant™ group (0.35 vs. 0.03 respectively), but this was not significant (p = 0.644). In non-DPN subjects a 40% greater reduction in severity was observed in Celliant™ subjects (p = 0.230). Non-DPN Celliant™ subjects reported 34% more reduction in interference compared with controls (p = 0.760).

**Figure 3:**
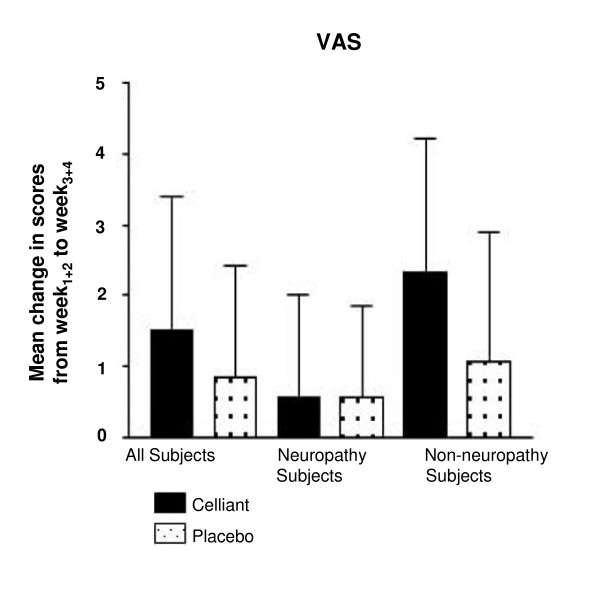
**Results of the VAS**. The difference between mean W_1+2 _and mean W_3+4 _scores is depicted. Solid bars report Celliant™ and stipled bars report control subjects.

The Visual Analog Scale (VAS) rated foot pain from 0 (none) to 10 (worst possible) during the previous week. The entire Celliant™ group reported 45% greater reduction in pain compared to controls (p = 0.127; Figure 3). Changes between W_1+2 _and W_3+4 _VAS pain scores did not vary significantly between Celliant™ and control DPN subjects (0.10 compared to 0.00, p = 0.849) (Figure 3). In the non-DPN group, Celliant™ subjects exhibited 54% more reduction in pain compared to controls (p = 0.060).

**Figure 4:**
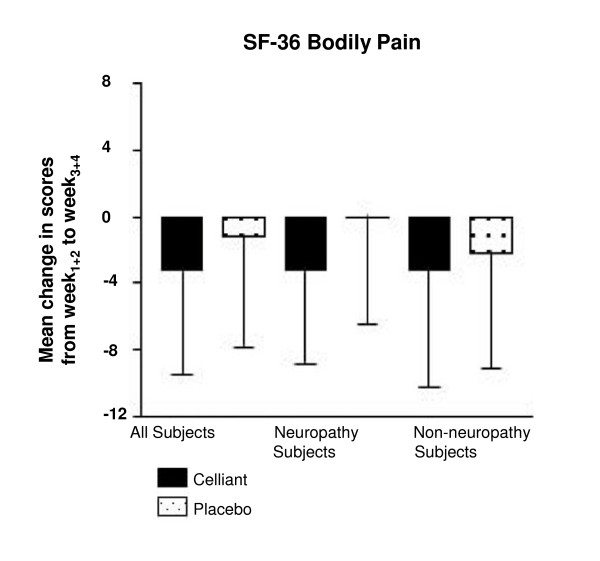
**Results of the SF-36 Bodily Pain**. The difference between mean W_1+2 _and mean W_3+4 _scores is depicted. Solid bars report Celliant™ and stipled bars report control subjects.

The SF-36 questionnaire has 10 categories measuring health and wellness. The bodily pain score measures a subject's attitude towards pain. Higher scores reflect less pain and lower scores more. Reduced pain correlates with negative [W_1+2 _- W_3+4_] results. Figure 4 shows the Celliant™ group had 62% more improvement compared to controls (p = 0.058). In DPN subjects, there was 99% greater improvement in the pain score with Celliant™ compared to controls (p = 0.109). For non-DPN subjects, pain improvement with Celliant™ was 29% greater compared to controls (p = 0.275).

## Discussion

This is the first trial assessing the impact of optically modified PET garments on pain. The pain questionnaires employed have been validated in previous studies [1-7], and were modified only by asking subjects to consider foot pain in their replies (except for the SF-36). Although a placebo effect was observed for most questions (controls reported improvement in 7 out of 9, 3 significantly), more reduction in pain was reported by subjects wearing Celliant™. The response to MPQ question III, in particular, showed significantly greater reduction in pain for Celliant^® ^compared to controls. In the DPN subgroup, two questions failed to show greater improvement with Celliant™ compared to placebo: MPQ questions Ib and Ia+b. These questions employ multiple complex scales and are designed more to measure sensory and affective aspects of pain rather than intensity. For all subjects, only question Ib on the MPQ did not display results favouring the Celliant™ group. Similarly, the BPI pain interference question does not address pain intensity and in the DPN subgroup, more improvement was found in the control group (p > 0.566). Table 3 shows the aggregate result for all pain questions.

**Table 3:** Results of pain questions

**Question**	**All Subjects**	**DPN subgroup**	**Non-DPN subgroup**
**McGill Ia**	+	+	+

**McGill Ib**	-	-	-

**McGill Ia+b**	+	-	+

**McGill II**	+	+	+

**McGill III**	+**	+	+

**BPI Pain Severity**	+*	+	+

**BPI Pain Interference**	+	-	+

**VAS**	+	+	+*

**SF-36 Bodily Pain**	+*	+	+

(+) Celliant™ showed greater improvement; (-) Controls showed greater improvement

** p < 0.05, *< 0.10

Overall the data reported show more improvement in pain reported by subjects wearing the Celliant^® ^socks compared to the controls. The lack of statistical significance for the differences in results with most of the questions may be due to the relatively low number of subjects in this pilot study as well as a lack of homogeneity in the subjects.

In our study each questionnaire was administered twice before and after dispensing the study garments with the results averaged, in the hopes of increasing the precision of the pain assessments. This might skew the data if the therapeutic effect of the Celliant™ socks changes with time – either increasing or decreasing. In future studies employing larger number of subjects this methodological problem should be avoided by administering each set of pain questionnaires only once.

In general, non-DPN subjects showed more sensitivity to the beneficial effect of Celliant™ than subjects with DPN. Assuming the effect of Celliant™ on tissue is relatively localized, one might expect less of an effect to be seen in neuropathy, as only a portion of the diseased neuron fibers are in close proximity to the plantar aspect of the socks, and thus likely subject to the effect of the modified fabric.

This raises the question of what mechanism could account for the apparent beneficial impact of optically modified fiber garments. Two unpublished studies, one in healthy subjects and one in diabetics, demonstrated significant increases in transcutaneous oxygen tensions in the skin of the hands and feet when Celliant™ garments were worn compared to placebo garments (Lavery LA, 2003; McClue GM and Lavery LA, 2003). The increased oxygen tensions were observed by 10 minutes and persisted during repeated measurements over 60 minutes. The increase in healthy subjects ranged from 10 to 24%; diabetic subjects showed an average increase of 10%. It is conceivable that some interaction of the Celliant™ particles with light increases reflection or transmission of light in the visible or near infrared portion of the spectrum into the skin, leading to vasodilation of the microcirculation and enhanced perfusion of tissue, which plausibly could ameliorate some causes of chronic pain. Alternatively, the enhanced illumination of the skin and underlying tissues could influence the biologic activity of endogenous chromophores (cytochromes, flavins, and poryphyrins) involved in energy metabolism in a manner leading to anti-inflammatory or anti-nocioceptive effects.

A large body of evidence suggests that short periods of illuminating skin, tissue, and cells with visible or infrared light has positive effects on pain, injury recovery, and wound healing. A number of studies have looked at joint pain such as temporomandibular joint pain [8], finding that near infrared light (810 nm) appears to reduce pain compared to sham illumination regimens. A meta-analysis of 20 trials employing laser therapy for chronic joint disorders found that when sufficiently intense light was employed, such therapy had a direct anti-inflammatory effect on the joint capsule [9]. A study of the effects of infrared (950 nm) on sural nerve conduction showed significant impact of illumination on nerve conduction velocity and negative peak latency compared to sham illumination [10]. Several studies on diabetic neuropathy showed a favourable impact of intermittent illumination with infrared at 890 nm on sensation and pain [11,12]. Low level illumination of joints affected by osteoarthritis by infrared diodes emitting at 890 nm has also been reported as effective for alleviating pain, and the effect has been postulated to be related to stimulation of constitutive nitric oxide synthetase [13]. Low intensity laser therapy at 810–820 nm combined with exercise regimens has been shown to benefit patients with chronic back pain and Achilles tendonopathy [14,15]. Several studies using animal models of wound healing or cell cultures have examined the effects of short exposures to red (e.g., 632 nm, 670 nm) or infrared light (e.g., 830 nm), finding wound healing to be significantly accelerated or increased expression of genes and proteins associated with proliferation [16-21].

Previous studies generally entailed short illumination periods of a few minutes at intensities of 1 to 20 Joules/cm which are much higher than the presumptive low intensity optical effects of Celliant™ garments. Our subjects were wearing socks under ambient light conditions and often shoes. Past demonstrations of interactions between tissues and external light, nonetheless, support the possibility that Celliant™'s effect is due to prolonged exposure of underlying structures to an altered electromagnetic environment. Given the putative anti-inflammatory effects of infrared light, the ability of longer wavelengths to penetrate more deeply, and the likelihood that Celliant™ particles significantly reflect and scatter infrared light, plausibly the Celliant™ effect is mediated by perturbations in the infrared portion of the spectrum. Conceivably, but we think unlikely, the Celliant™ effect may be due to higher skin temperatures resulting from more efficient reflection of infrared energy, but this requires further investigation. We are now planning further studies employing thermography and hyperspectral imaging of skin blood flow to further characterize the effects of wearing Celliant™ garments.

## Conclusion

The data from this pilot study suggests that wearing Celliant™ fabric socks may reduce the pain associated with chronic foot disorders. Future studies in larger numbers of subjects looking at other chronic pain conditions such as carpal tunnel syndrome and knee arthropathies are warranted as well as attempts to elucidate the mechanism by examining the influence of the modified garments on tissue perfusion, temperature, oxygen levels, and inflammation.

## Competing interests

The authors declare that they have no competing financial interests. Hologenix, LLC funded the study (see acknowledgements) and manufactures the garments employed in this study.

## Authors' contributions

RY participated in the design of the study, carried out subject recruitment and data collection, took part in the statistical analysis, and helped draft the manuscript. IG conceived of the study, took part in its design and coordination, led the statistical analysis, and helped draft the manuscript. All authors read and approved the final manuscript.

## Pre-publication history

The pre-publication history for this paper can be accessed here:


